# Language/Culture Modulates Brain and Gaze Processes in Audiovisual Speech Perception

**DOI:** 10.1038/srep35265

**Published:** 2016-10-13

**Authors:** Satoko Hisanaga, Kaoru Sekiyama, Tomohiko Igasaki, Nobuki Murayama

**Affiliations:** 1 Division of Cognitive Psychology, Faculty of Letters, Kumamoto University 2-40-1, Kurokami, Chuo-ku, Kumamoto, 860-8555, Japan; 2 Department of Information Technology on Human and Environmental Science, Graduate School of Science and Technology, Kumamoto University, 2-39-1, Kurokami, Chuo-ku, Kumamoto, 860-8555, Japan

## Abstract

Several behavioural studies have shown that the interplay between voice and face information in audiovisual speech perception is not universal. Native English speakers (ESs) are influenced by visual mouth movement to a greater degree than native Japanese speakers (JSs) when listening to speech. However, the biological basis of these group differences is unknown. Here, we demonstrate the time-varying processes of group differences in terms of event-related brain potentials (ERP) and eye gaze for audiovisual and audio-only speech perception. On a behavioural level, while congruent mouth movement shortened the ESs’ response time for speech perception, the opposite effect was observed in JSs. Eye-tracking data revealed a gaze bias to the mouth for the ESs but not the JSs, especially before the audio onset. Additionally, the ERP P2 amplitude indicated that ESs processed multisensory speech more efficiently than auditory-only speech; however, the JSs exhibited the opposite pattern. Taken together, the ESs’ early visual attention to the mouth was likely to promote phonetic anticipation, which was not the case for the JSs. These results clearly indicate the impact of language and/or culture on multisensory speech processing, suggesting that linguistic/cultural experiences lead to the development of unique neural systems for audiovisual speech perception.

In face-to-face speech perception, what we hear is influenced by visual information of articulatory movements. One striking example is the McGurk effect^1^ in which discrepant auditory and visual information results in a fused perception. For example, auditory /ba/ combined with visual mouth movements for /ga/ is often perceived as ‘da’ or ‘tha’. While this audiovisual (AV) integration of speech cues is robust for adult native speakers of English^2^,^3^,^4^ and other European languages^5^,^6^,^7^,^8^, the size of the McGurk effect is known to be much smaller in Japanese perceivers^9^,^10^,^11^. A difference in the size of the McGurk effect has also been observed between children and adults in European language participants^1^,^7^,^11^ with reports indicating that children rely more on auditory information than adults in face-to-face speech perception^12^. Interestingly, linguistic and/or cultural differences emerge at some point during development. The degree of visual influence was low and similar for Japanese and English 6-year-olds, and increased with age for English language participants, but remained the same for Japanese participants^11^. In these developmental transitions that differ between Japanese and English, noticeable differences in response time (RT) were also found. In unimodal speech perception, native English speakers develop faster visual-only speech perception (lipreading) than auditory-only (AO) speech perception (hearing) while native Japanese speakers continue to show equivalently fast hearing and lipreading. Such a transition may be crucial to the visual influence on auditory speech processing. The reason why Japanese native speakers are less affected by visual speech is not clear, but some linguistic and/or cultural factors could play a role. As a potential linguistic factor, for example, the Japanese language may be characterized by its less informative visual lipread information^13^ compared with English^14^,^15^.

In agreement with the smaller McGurk effect in Japanese, it has also been reported that Japanese are subject to less visual influence than the Western subjects in AV emotion perception^16^. When an audiovisually incongruent emotion was presented, Japanese perceivers were more accurate in reporting vocal emotion by ignoring facial expressions than Dutch perceivers. The Japanese may have a tendency to rely more on voice than face in AV speech-related decision tasks. In recent studies in cultural psychology, Eastern-Western differences in attention have been postulated. When recognizing facial expressions, Eastern people value the eye region, whereas Western people value the mouth region^17^,^18^. If such cultural differences are also present during AV speech perception, Japanese and English perceivers may pay attention to different regions when perceiving AV speech.

Previous studies have examined ERP for AV speech perception in English^19^,^20^, Dutch^21^, French^22^, and Finnish^23^,^24^. These studies have shown that the influence of visual speech is apparent in relatively early ERP peaks such as the N1 (negative 100 ms peak) and P2 (positive 200 ms peak). N1/P2 amplitude and latency decrease in the AV condition compared with the AO condition^19^,^20^,^23^,^24^. A reduction in the N1 amplitude and latency are observed not only for speech events but also for non-speech events where an anticipatory visual motion precedes the sound^21^,^25^,^26^. A few studies have suggested that this early integration at N1 is related to the temporal and spatial features of the stimuli^25^,^26^. In contrast, the P2 reduction is often thought to be at a phonetic, semantic, or associative level^23^, and, in fact, is content-dependent such that the amplitude reduction of P2 was larger for incongruent than congruent AV stimuli^21^. A later integration process has been also indicated by a magnetoencephalography/functional magnetic resonance imaging study^27^. Based on those reports, we hypothesized that the later integration process represented by P2 is related to cross-linguistic differences because the difference might be at the phonetic level.

This study aimed to examine how the cultural and/or linguistic environments may affect perceptual processing style in AV speech perception. We used three indices: RT, ERP, and eye-tracking measurements. First, RT was measured to examine perceptual speed for AO and AV congruent speech (Experiment 1). We predicted that AV information would produce faster speech perception than AO information in English speakers, but that Japanese would not benefit from visual information^11^. Second, ERP was assessed to investigate neurophysiological processes for audiovisually congruent speech (Experiment 2). We focused on the N1 and P2 components to reveal any group differences. Third, eye gaze while perceiving AV speech was recorded (Experiment 3) to examine the differences in attentional bias between Western and Eastern participants. We hypothesized that gaze differences would be observed in AV speech perception based on the results of prior studies^17^,^18^. By combining these techniques, we aimed to uncover the differences in AV speech perception between Japanese and English speakers.

After clarifying group differences in these three measures, we further investigated whether an instruction to focus on the mouth could increase the visual influence on the Japanese speakers’ audiovisual speech perception (Experiment 4). To measure the size of the visual influence behaviourally, we used incongruent stimuli (e.g., audio /ba/ + visual /ga/) in addition to congruent stimuli and the size of the visual influence was measured in a McGurk effect paradigm.

## Results

The participants were presented movie clips of two female talkers (a native Japanese and a native English speaker) articulating /ba/ and /ga/ syllables for a syllable identification task. There were two conditions (AO & AV) in Experiment 1 (RT) and 2 (ERP), and only the AV condition in Experiment 3 (eye-tracking). In Experiment 4, in addition to the audiovisually congruent speech stimuli used in Experiments 1–3, incongruent stimuli (e.g. auditory /ba/ combined with visual /ga/) were also presented to examine the size of the visual influence in a McGurk effect paradigm.

### Experiment 1: Differences in response time

Each participant was asked to make a perceptual decision about whether the presented syllable was ‘ba’ or ‘ga’ by pressing one of the two buttons. They were instructed to look at and listen to each speaker. The percentage of correct responses was almost perfect (99.57% for the AO condition and 100% for the AV condition in JSs, and 99.69% for the AO condition and 100% for the AV condition in ESs). Due to this ceiling effect in accuracy, only RTs were used as an index of performance.

**Figure 1:**
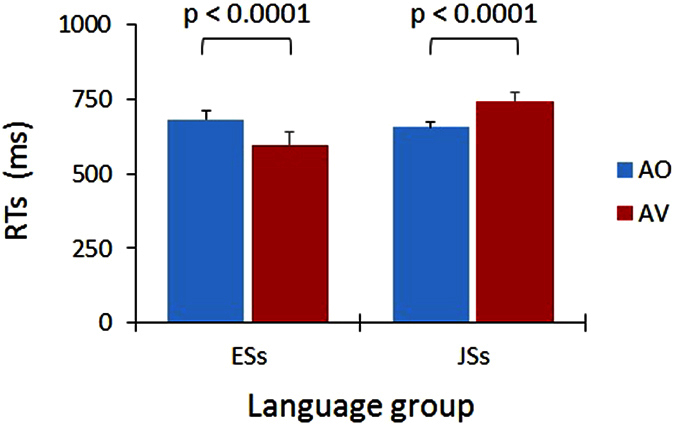
RTs of ESs and JSs in AO (blue) and AV (red) conditions. While congruent visual speech shortened RTs for ESs (shorter RTs for the AV condition than the AO condition), it delayed the RTs for JSs. Error bars show standard errors. RT: response time; ESs: native English speakers; JSs: native Japanese speakers; AO: audio-only; AV: audiovisual.

Table 1 shows the mean RT for each group, in each condition, and for each stimulus (specified by the speaker and syllable). A four-way analysis of variance (ANOVA) (language group × condition × talker × syllable) on RTs was performed. As predicted, the interaction of language group × condition was significant (F (1, 37) = 21.18, p < 0.0001, ηp^2^ = 0.36). Detailed analyses of the simple effects for this interaction revealed a significant simple main effect of condition on ESs (F (1, 37) = 8.95, p < 0.005, ηp^2^ = 0.19) and JSs (F (1, 37) = 12.95, p < 0.001, ηp^2^ = 0.25). A post hoc Tukey test showed that RTs for the AV condition were significantly shortened compared with AO in ESs (p < 0.0001, r = 0.65; r: effect size), whereas RTs for the AV condition were significantly prolonged in JSs (p < 0.0001, r = 0.58). Thus, additional visual speech showed opposing effects in the two language groups (Fig. 1).

Other significant terms were the main effect of syllable (F (1, 37) = 7.85, p  < 0.01, ηp^2^ = 0.18) and the interaction of condition × syllable (F (1, 37) = 4.95, p < 0.05, ηp^2^ = 0.12). For the condition × syllable interaction, the simple main effect of syllable was significant in the AV condition (F (1, 74) = 12.80, p < 0.001, ηp^2^ = 0.15). A post-hoc Tukey test indicated that RTs for syllable /ba/ were faster than those for /ga/ only in the AV condition (p < 0.0001, r = 0.51), suggesting that visual /ba/ carries more information than /ga/28. No other effects were significant: main effects of language group (F (1, 37) = 2.63, p = 0.11, ηp^2^ = 0.07), condition (F (1, 37) = 0.00007, p = 0.99, ηp^2^ = 0.03), and talker (F (1, 37) = 0.91, p = 0.35, ηp^2^ = 0.02), as well as the other interactions.

### Experiment 2: ERP modulations by visual speech

**Figure 2:**
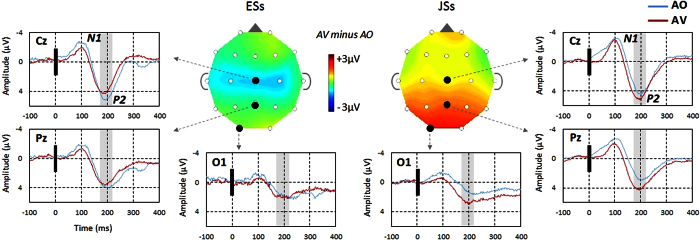
Grand average ERPs time-locked to the audio onset for the three electrodes (Cz, Pz, and O1) in the AO (blue) and AV (red) conditions. The black vertical line indicates the audio onset. The left panels are for ESs (N = 8) and right panels for JSs (N = 12). The coloured topographic map shows the differences in amplitude between the two conditions (AV−AO) for the 170–220 ms time window (depicted by the grey background on each graph). The colour distribution of the two maps clearly demonstrates the differences between ESs and JSs for the central and posterior electrodes (see Table 2 for statistics). ERP: event-related potential.

The task in the ERP experiment was the same as that in the RT experiment except that no overt response was requested. We analysed ERP latency and amplitude by focusing on the difference between the two conditions (AV−AO) to quantify the visual influence. Fig. 2 shows the averaged ERP data for each electrode we analysed. In addition, individual ERPs at the Cz electrode for the AO and AV conditions are shown in Fig. S1. As described in the Supplementary Information, the group difference was not driven by outliers in either latency or peak amplitude for N1 and P2.

Latency: We examined the latency of N1 and P2 at the vertex electrode (Cz) because the auditory evoked potential reaches its maximum near the vertex^29^. Different group characteristics were observed for the P2, but not N1 latency reduction. For N1, the Mann–Whitney U test revealed no significant difference in latency reduction between the two language groups (U = 39.5, p = 0.54, r = 0.14; r: effect size). For P2, a significant group difference was found (U = 15, p < 0.05, r = 0.56). JSs showed less P2 latency reduction (AV−AO; M = −2.5 ms) compared with ESs (M = −19 ms).

Amplitude: First, we examined the amplitude at the vertex electrode (Cz). Similar to the latency analysis, different group characteristics were found in the P2 (U = 8, p < 0.005, r = 0.65) but not N1 peak amplitude difference (U = 42, p = 0.67, r = 0.10). Visual information increased the P2 amplitude in JSs, while the opposite effect was observed in ESs. Secondly, we examined the different group characteristics of the peripheral regions including C3, C4, P3, Pz, P4, O1, and O2. Analogous to the vertex peak analyses, we set two 50 ms time windows: the N1 window (70–120 ms from audio onset) and P2 window (170–220 ms from audio onset). As summarized in Table 2, the group differences were observed to be significant in the P2 window for every electrode, but there were no significant differences in the N1 window except for the C3 electrode.

Considered together, both latency and amplitude generally indicated that group differences were significant in P2 (and the 170–220 ms window), but not N1 (and the 70–120 ms window).

### Multiple regression analyses for behavioural RT

For those who participated in both Experiments 1 and 2, we examined the relationship between RT and ERP (at the vertex electrode, Cz) by focusing on the visual influence (AV−AO). The multiple regression analyses were conducted with the RT differences (AV−AO) as the outcome, and five predictor variables were used: language group, N1 peak amplitude reduction, P2 peak amplitude reduction, N1 latency reduction, and P2 latency reduction. To find significantly contributing predictors, the backward stepwise method was used. The matrix of simple correlation coefficients is shown in Table 3. The most optimal regression model found in the analysis revealed that two variables significantly predict RT differences; language group (standardized partial β = −0.43, t = −2.17, p < 0.05) and P2 peak amplitude (standardized partial β = 0.43, t = 2.17, p < 0.05). The general linear model with these two variables reasonably fits the data, with the adjusted R^2^ = 0.55, F (2, 17) = 12.80, p < 0.001. The results indicate that the impact of visual speech on RTs is significantly predicted by the participant’s native language and P2 amplitude reduction.

**Figure 3:**
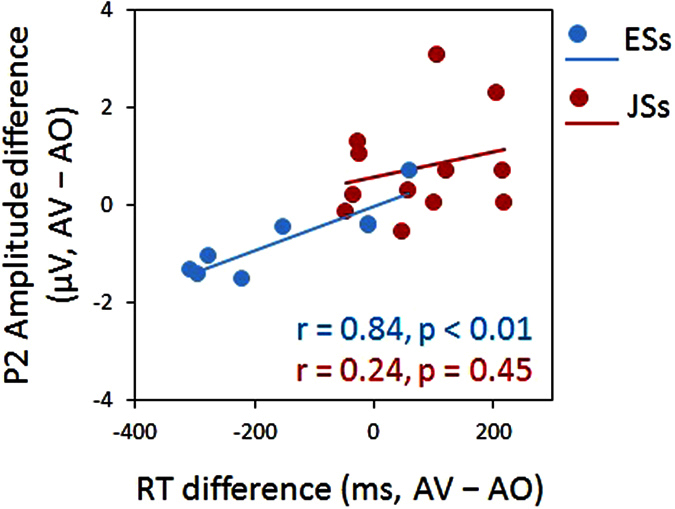
Plot between RT difference and ERP P2 amplitude reduction due to visual speech. Data are plotted for those who participated in both Experiments 1 and 2. Correlation analyses revealed that only ESs showed a significant correlation between the two measures.

According to the obtained model, we calculated the correlation coefficients between RT reduction and P2 peak amplitude reduction for each group (ESs: r = 0.84, p < 0.01; JSs: r = 0.24, p = 0.45), finding that the P2 peak amplitude correlates with RT only in ESs (Fig. 3).

### Experiment 3: Differences in gaze bias

**Figure 4:**
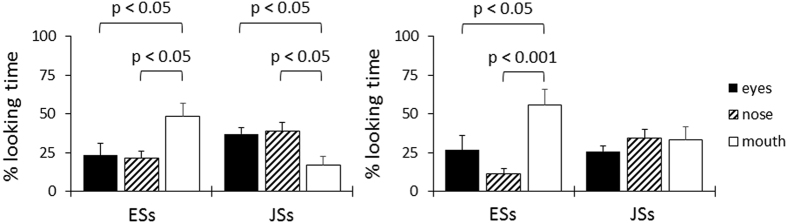
Percentage of looking time for each AOI (eyes, nose, and mouth) for each language group (left panel; TW1, from movie onset to audio onset, right panel; TW2, from audio onset to mouth closure). The error bars show standard errors. AOI: area of interest; TW1: time window 1; TW2: time window 2.

In a third experiment, we specifically explored what visual information influences auditory speech processing. To analyse the data, we set two time windows: one from the movie onset to audio onset (TW1) and the other from audio onset to mouth closure (TW2). We assumed visual information that influences speech perception qualitatively differed between these two periods. We created three specific areas of interest (AOI): eyes, nose, and mouth (defined by a rectangle) as well as the entire face (excluding the hair and ears). We calculated the proportion-of-total-looking-time (PTLT), which is the proportion of looking time that participants spent at each AOI on the entire face. The PTLT scores were analysed using a three-way ANOVA (language group × time window × AOI), and, as shown in Fig. 4, the results indicated a gaze bias to the mouth only in ESs for both time windows, and that JSs had an eye-and-nose bias in TW1 and no bias in TW2. There were significant interactions of language group × AOI (F (1.62, 43.64) = 4.48, p < 0.05, ηp^2^ = 0.14), time window × AOI (F (1.65, 44.57) = 8.88, p < 0.005, ηp^2^ = 0.25), and language group × time window × AOI (F (1.65, 44.57) = 3.47, p < 0.05, ηp^2^ = 0.11). The simple main effect of the AOI was significant in the ESs, but not in the JSs (ESs: F (2, 24) = 4.80, p < 0.05, ηp^2^ = 0.29; JSs: F (2, 30) = 0.56, p = 0.58, ηp^2^ = 0.04). A post-hoc Tukey test indicated that in the ESs the PTLT scores for the mouth area were higher than the eye (p < 0.005, r = 0.57) and nose areas (p < 0.0001, r = 0.67). For the language group × time window × AOI interaction, the post-hoc Tukey test revealed that in TW1 and TW2 the PTLT scores for the mouth area were higher than the eye (TW1; p < 0.05, r = 0.47, TW2; p < 0.05, r = 0.52) and nose areas (TW1; p < 0.05, r = 0.49, TW2; p < 0.0005, r = 0.68) in the ESs. In the JSs, the PTLT scores for the mouth area were lower than the eye (p < 0.05, r = 0.32) and nose areas (p < 0.05, r = 0.34) in TW1, but not TW2. As shown in the left panel of Fig. 4, the mouth area was well focused on before audio onset in ESs (48.47%), which is strikingly different from the fixation patterns in JSs (17.05%).

### Experiment 4: Effect of gaze manipulation on AV speech perception in JSs

Based on the gaze results in Experiment 3, we investigated whether the characteristics found in JSs, in which visual speech did not benefit speech perception, were modifiable by an instruction to focus on the mouth. To test this, we used a McGurk effect paradigm. We presented incongruent AV stimuli (AVi) in addition to the congruent AV stimuli (AVc) used in the previously detailed three experiments. The participants were asked to decide what they perceived in one of two instruction conditions. The ‘instructed group’ were asked to pay attention to the mouth region during the test block while the ‘non-instructed group’ had no specific instructions regarding attention. Prior to the test block with the instruction manipulation, each group underwent a control block in which no specific instruction on attentional focus was given, the group was simply asked to ‘look at the speaker, listen to the sound, make a decision as to whether it was “ba”, “da”, or “ga”, and press one of the three buttons’.

**Figure 5:**
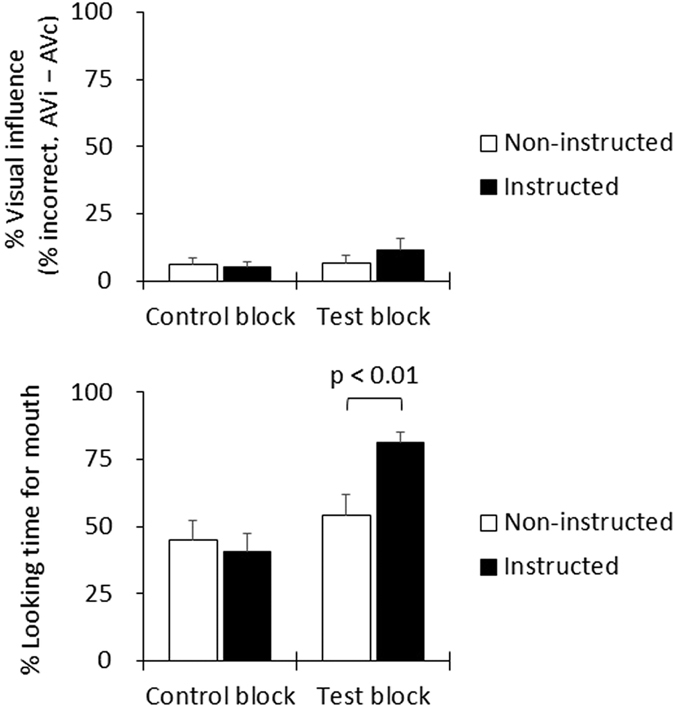
The upper panel shows the size of visual influence (difference in % visual influence, AVi−AVc) in each block. The lower panel shows % looking time for the mouth in each block (from the movie onset to the audio onset). As indicated in the test block, the instruction to look at the mouth increased looking time for the mouth (lower panel), however, the instruction did not increase the visual influence (upper panel). AVi: audiovisually incongruent stimuli; AVc: audiovisually congruent stimuli.

The size of the visual influence was calculated as the difference in (auditory) error rate between the incongruent and congruent conditions (AVi−AVc) for each block. A two-way ANOVA (group × block) revealed that the two groups performed similarly irrespective of the difference in instructions in the second block (group; F (1, 34) = 0.28, p = 0.60, ηp^2^ = 0.0082, block; F (1, 34) = 2.14, p = 0.15, ηp^2^ = 0.06; interaction of group × block (F (1, 34) = 1.46, p = 0.24, ηp^2^ = 0.04) (upper panel on Fig. 5). As a result, the instruction to ‘look at the mouth’ did not increase the visual influence.

For fixation data, we focused on a time window before the audio onset. Looking time for the mouth region before audio onset was analysed using a two-way ANOVA (group × block). This analysis showed a significant interaction between group × block (F (1, 34) = 9.28, p < 0.005, ηp^2^ = 0.21) indicating that there was no group difference in the control block (F (1, 34) = 2.09, p = 0.16, ηp^2^ = 0.06) and a significant difference in the test block. In the test block, the instructed group fixated on the mouth longer than the non-instructed group (F (1, 68) = 8.40, p < 0.01, ηp^2^ = 0.11), indicating that the instruction was successfully followed (lower panel on Fig. 5). Taken together, the instruction to focus eye gaze on the mouth increased the time spent looking at the mouth, but did not enhance the small visual influence seen in JSs.

## Discussion

In this study, we aimed to clarify how Japanese and English speakers differ in processing AV speech as a speech event proceeds. To elucidate such dynamic processes in terms of both perceptual and cortical aspects, we measured RT (Experiment 1), ERP (Experiment 2), and eye gaze (Experiments 3 and 4). In particular, we investigated whether visual speech facilitates the auditory speech processing of Japanese perceivers as reported in European language perceivers^19^,^20^,^21^,^22^.

As a result, we found that additional visual speech increased the efficiency of auditory speech processing in English perceivers, while it added an additional processing load to Japanese perceivers as revealed by ERP and RT. As we hypothesized, ERP group differences were limited to P2, and most prominently observed for P2 amplitude. The amplitude was smaller for ESs and larger for JSs in the AV condition than in the AO condition. Moreover, the P2 amplitude reflected the group differences in RT. The P2 amplitude reduction in ESs may be related to visual anticipation of phonetic information^21^,^27^. JSs did not show reduced amplitudes for the AV condition, but actually showed increased amplitudes. Thus, the auditory-visual interplay is completely different between these language groups. We interpret the present results as follows. Based on the results of Experiment 3, ESs may have already paid attention to the mouth movement before auditory speech occurred, so the auditory information was easy to process for ESs based on visually induced anticipation. In contrast, the JSs demonstrated minimal, if any, sampling of visual speech information before the audio onset for syllable judgment. This ineffectiveness may have elicited an additional cognitive load (similar to the effect of a dual task) reflected by an increase in the amplitude in the AV condition compared with the AO condition, in contrast to ESs. In light of the effect of visual information on RT in Experiment 1 (Fig. 1) and the result of multiple regression analysis (Experiment 2), the AV speech interplay had a facilitating effect for ESs, but an interfering effect for JSs, which was reflected in an earlier ERP component, P2 amplitude. The effect of language/culture may be represented in P2, which is related to audiovisual interplay at a phonetic level^21^,^27^, but not in N1, which is related to audiovisual interplay at a spatial and temporal level^25^,^26^.

As noted above, previous studies have confirmed the P2 amplitude reduction due to additional visual speech for English-speaking participants^19^,^20^, consistent with the ESs in the present study. However, a few recent studies on French-speaking participants reported equivalent P2 amplitudes for AV and AO speech processing^30^,^31^. Although this may be related to the linguistic/cultural characteristics of French-speaking participants, it may also be due to differences in the experimental setting. For example, these French studies used face-to-face live speech stimuli compared with recorded speech used in other studies, and the baseline period for electroencephalogram (EEG) analysis was also different (from 500 ms to 400 ms prior to audio onset) from other studies (a few hundred ms-period immediately before audio onset). In any case, the Japanese participants in the present study showed an increased P2 amplitude, which was different from the French participants in the above studies^30^,^31^.

The present study gives some clues to understanding language and/or cultural differences in the McGurk effect, which has been shown to be weaker in Japanese perceivers than English perceivers^9^,^10^,^11^. This study demonstrated that JSs did not anticipate auditory phonetic counterparts by utilizing the preceding visual preparatory movement for articulation. Thus, the reason why Japanese are less susceptible to the McGurk effect may be that they extract less visual information before auditory speech starts. This processing style in Japanese for audiovisual stimuli could also affect emotion perception. Tanaka *et al.*^16^ indicated that Japanese people could ignore facial expressions, but could not ignore voice in audiovisual emotion judgment^16^. Japanese may choose to concentrate on the content in auditory information when they perceive audiovisual speech-related information.

Why do Japanese not use the visual articulatory (preceding the sound) information of the mouth in phonetic processing? Linguistic factors, such as less informative lip-read information might be responsible for this phenomenon. English consonants can be divided into the five or six consonants groups by lipreading^14^,^15^, while Japanese consonants are divided into only three groups^13^. Therefore, the usefulness of visual information for speech perception is different between these languages. These results are consistent with the results of Hazan and her colleagues^32^ who showed that Japanese would not benefit from additional visual speech in second language learning. In this way, linguistic factors could affect the AV processing style. As Experiment 4 revealed, the processing style of Japanese speakers seems to not be affected by temporary focus of the eye gaze on the mouth. Perhaps what matters is the accumulation of visual attention on the mouth during language acquisition in the early stages of life. The present study was not designed to determine the cause of the group differences, but it did reveal that the tendency of JSs not to look at the mouth may be based on the linguistic features of Japanese, in which mouth movement provides less information.

The areas where visual attention is focussed might be related to other cognitive functions. That is, Eastern people value the eye region, whereas Western people value the mouth region. This has been demonstrated in facial expression judgment tasks^17^,^18^. Although our experiments were different from the facial expression judgment tasks, the previous findings may partly explain our results that revealed only ESs showed a gaze bias for the mouth. On the other hand, JSs did not show a gaze bias for the mouth even when the task was speech perception for which the mouth could provide salient information. These striking differences seem to be consistent with the language and/or cultural gaze differences described above, especially the fact that JSs looked at the mouth much less than the eyes and nose before the onset of auditory speech.

The present study demonstrates that neural processes for audiovisual speech processing are not universal. According to the previous behavioural finding that inter-language differences appear between 6 and 8 years of age^11^, the differences in neural processes may be fostered by linguistic and/or cultural experiences at an early school age. After language acquisition, people acclimatize themselves to the linguistic/cultural environment, which affects AV speech processing. Our neural and behavioural characteristics in AV speech perception may be defined by our linguistic/cultural background.

## Materials and Methods

### Participants

Sixteen ESs (10 males and 6 females; mean age = 20.6 years) and 23 JSs (8 males and 15 females; mean age = 20.8 years) were recruited for Experiment 1 (RT). Among the participants recruited in Experiment 1, eight ESs (four males and four females; mean age = 20.1 years) and twelve JSs (five males and seven females; mean age = 21 years) participated in Experiment 2 (ERP). Thirteen ESs (nine males and four females; mean age = 21.77 years) and sixteen JSs (seven males and nine females; mean age = 21.31 years) were recruited for Experiment 3 (eye-tracking). Of the participants in Experiment 3, five ESs and fourteen JSs were naive to this study. In Experiment 4, 36 young Japanese participants were recruited. Half of them were assigned to the ‘instructed’ group and the rest ‘non-instructed’ group. The former group was instructed to attend mouth region during the task, and the latter group had no specific instruction about attention. All participants were healthy university students living in Kumamoto. All English native speakers were visiting students, and had lived in Japan for less than 3 months (mean = 1.83 months). No JSs had any experience of staying abroad for longer than 3 months. They were all right-handed and had normal hearing and normal or corrected-to-normal vision.

### Ethics Statements

The methods in all experiments were carried out in accordance with the Declaration of Helsinki. The experimental procedure in this study was approved by the Ethical Committee of Kumamoto University Graduate School of Science and Technology. Written informed consent was obtained from each participant.

### Stimuli and Procedure

Stimuli were prepared by video-recording two female talkers (a native English and a native Japanese talkers) while each of them uttered /ba/ and /ga/. The movie clips were edited for AV and AO conditions. The AV stimuli consisted of matching auditory and visual speech. At the movie onset, the talker’s face appeared with the mouth closed in a neutral position. The onset of auditory speech was aligned at 900 ms from the movie onset. The duration of the auditory speech was approximately 300 ms. As a characteristic of natural speech, the talker’s mouth in the AV stimuli drastically changed from approximately 400 ms before sound onset as a preparatory movement. The AO stimuli contained the same auditory speech as in the AV stimuli, but the image was replaced by a black video with a white fixation point at the centre. The video was digitized at 29.97-frames per s at 640 × 480 pixels. Sound was digitized using a 16 bit 44.1 kHz resolution and was stored in stereo. The auditory stimuli were presented through a loudspeaker at 65 dB. To mask fan noise, a weak auditory noise (a band noise of 300–12000 Hz) was presented at a signal-to-noise ratio of 15 dB. This SN ratio was confirmed to have no influence on speech perception accuracy in our previous study^11^. The noise started approximately 10 seconds before the presentation of stimuli and continued until the end of the experimental block. The visual stimuli were presented at the centre of the monitor, with the visual angle of the faces at 7.2° (vertical) and 5.1° (horizontal). The loudspeaker was placed above the monitor.

Experiment 1 consisted of 40 trials in which each of the four stimuli (/ba/ and /ga/ articulated by a Japanese and English speaker) were presented 10 times. With the two different conditions (AO and AV), there were a total of 80 trials. The presentation order was counterbalanced across participants. The participants were instructed to look at the display, listen to the sound, make a decision whether it was ‘ba’ or ‘ga’, and press one of two buttons. Response time was measured as the time from the audio onset to the button press. The onset of the next stimulus was 1,500 ms after the button press.

In Experiment 2, participants were presented with 10 blocks of AV stimuli (each /ba/ and /ga/ for each speaker was presented 10 times per block, thus there was a total of 40 trials in a block) and 10 blocks of AO stimuli (also 40 trials per block). In total, there were 800 trials. The AO and AV blocks were alternated, and the presentation order was counterbalanced across participants. Each participant underwent two AV and two AO blocks on the first day and four AV and four AO blocks on each of the second and third days. The participants were instructed to look at the display, listen to the sound, and make a decision whether it was ‘ba’ or ‘ga’ without any overt response. This allowed motor-related potentials during ERP measurement to be ruled out. A trial consisted of a 2,000 ms stimulus period and a 1,500 ms blank interval.

In Experiment 3, each of the AV stimuli was presented six times per stimulus (total of 24 trials) in a fully pseudorandom fashion; we created two random sequences and the orders of the two sequences were counterbalanced across participants. Each participant’s eye position was calibrated using a five or nine-point routine prior to the experiment to ensure the positional validity of gaze measurements. The participant’s eye gazes were recorded by the eye tracker during the AV speech perception task. The procedure was similar to that of the Experiment 2 (ERP experiment); no overt responses were requested.

In Experiment 4, in addition to congruent AV stimuli, incongruent stimuli were used. Incongruent stimuli consisted of incongruent audio and visual information. The consonants were same as the stimuli in Experiments 1–3 except the addition of the /da/ sound, i.e., the visual or audio stimuli consisted of /ba/, /ga/, or /da/. Experiment 4 had three incongruent stimuli (auditory /ba/ combined with visual /ga/, auditory /da/ with visual /ba/, and auditory /ga/ with visual /ba/) and three congruent stimuli (auditory /ba/ combined with visual /ba/, auditory /ga/ with visual /ga/, and auditory /da/ with visual /da/). Each stimulus was repeated five times for a block (total of 60 trials through control and test blocks).

### Equipment

The auditory stimuli were presented through a loudspeaker (AIWA SC-B10) and the visual stimuli were presented at the centre of a 15-inch SONY SDM-S51 monitor. EEGs were recorded by a Neurofax EEG-1100 (Nihon Kohden, Tokyo) system using a scalp electrocap (International, Inc. Eaton, Ohio USA) with electrodes placed at Fp1, Fp2, F7, F3, Fz, F4, F8, T3, C3, Cz, C4, T4, T5, P3, Pz, P4, T6, O1, and O2 (10/20 system). Recording electrodes were referred to the linked earlobes (A1+A2), and the ground electrode was placed at Fpz. Electrode impedance was kept below 10 kΩ. The EEG signals were continuously amplified and digitized at a rate of 500 Hz/channel, and filtered online with a band pass of 0.53–300 Hz and a band stop of 60 Hz. A trigger signal (30 ms) was inserted on the audio track of each movie clip so that each trigger signal was synchronized with the onset of the auditory speech. The trigger signals were recorded on the 20th channel of the EEG, and the speech signals on the 21st channel to ensure synchronization. For eye tracking, the participant was seated in front of a monitor connected to a Tobii X120 eye tracker (screen refresh rate 60 Hz, eye-tracking sampling rate of 120 Hz).

### Data Analysis

Experiments 1 and 2 included a smaller data set that had been reported in a conference paper (ref. 33; eight Japanese and three English participants); however, the ERP analyses performed were not identical: In the previous paper, the artefact due to eye movement was not removed, and different time windows were used.

In Experiment 1, RTs were analysed using a four-way ANOVA (language group × condition × talker × syllable) with condition, talker, and syllable as repeated measures. If an interaction was significant, the simple main effects were analysed, and multiple comparisons were made by the Tukey test.

In Experiment 2, trials with the signal amplitudes exceeding 150 μV at any electrode from movie onset to 2,000 ms were automatically rejected because the response was considerably affected by eye movements. The mean rejection rate of the two conditions was 7.85%. The mean amplitude over the 200 ms before audio onset was taken as the baseline for all amplitudes for each trial. For latencies and peak amplitudes, we analysed N1 (approximately 100 ms after the audio onset) and P2 (approximately 200 ms after the audio onset) at Cz. The N1 peak was specified as the largest negative local maximum between 70–120 ms and the P2 peak as the largest positive local maximum between 170–220 ms. To analyse group differences in ERP latency and amplitude, we focused on the difference between the two conditions (AV−AO) as the degree of visual influence (reduction due to visual information), and this difference was tested using the Mann–Whitney U test. For peripheral regions, the amplitude analyses were conducted on mean amplitudes within 50 ms time windows for each electrode (seven levels: C3, C4, P3, Pz, P4, O1, and O2). We set the N1 (70–120 ms after audio onset) and P2 (170–220 ms after audio onset) windows. To examine the relationship between RTs and ERPs, multiple regression analyses were conducted with RT reduction (AV−AO) as the outcome variable. Five predictor variables were used: language group, N1 peak amplitude reduction, P2 peak amplitude reduction, N1 latency reduction, and P2 latency reduction.

In Experiments 3 and 4, the eye-tracking data were analysed within specific AOI to produce PTLT. In Experiment 4, the degree of visual influence was calculated by subtracting the error rate for congruent stimuli from that for incongruent stimuli. A correct response was defined in terms of the auditory component of a stimulus. The Greenhouse-Geisser correction was used where applicable.

## Additional Information

**How to cite this article**: Hisanaga, S. *et al.* Language/Culture Modulates Brain and Gaze Processes in Audiovisual Speech Perception. *Sci. Rep.* **6**, 35265; doi: 10.1038/srep35265 (2016).

## Supplementary Material

Supplementary Information

## Figures and Tables

**Table 1:** RTs (in ms) of ESs and JSs in AO and AV conditions for each stimulus (standard errors). RTs were measured as the time from the audio onset to the button press.

	ESs		JSs	
	AO	AV	AO	AV
E/ba/	662.51 (27.11)	561.57 (41.56)	660.71 (19.47)	729.04 (31.35)
E/ga/	687.54 (34.73)	626.79 (40.59)	647.93 (20.17)	761.93 (31.64)
J/ba/	687.87 (27.38)	591.95 (46.16)	653.95 (20.19)	731.97 (31.10)
J/ga/	694.95 (32.64)	602.67 (49.57)	664.59 (19.37)	755.36 (30.18)

E/ba/ indicates the /ba/-stimulus articulated by an English talker, J/ba/ indicates the /ba/-stimulus articulated by a Japanese talker. RT: response time; ESs: native English speakers; JSs: native Japanese speakers; AO: audio-only; AV: audiovisual.

**Table 2:** ERP reduction (AV–AO) in latency (ms) and amplitude (μV) for each electrode in each language group.

Peak latency (Cz)					Peak amplitude (Cz)				
	Median		U value	p		Median		U value	p
	ESs	JSs				ESs	JSs		
N1	−9.25	−15.25	39.5	0.54	N1	0.65	0.71	42	0.67
P2	−19.00	−2.50	15	*	P2	−0.74	0.51	8	**
**N1 window (70–120 ms) amplitude**					**P2 window (170–220 ms) amplitude**				
Electrode	Median		U value	p	Electrode	Median		U value	p
	ESs	JSs				ESs	JSs		
C3	0.03	0.32	22	*	C3	−1.25	0.88	14	**
C4	0.31	0.56	44	0.79	C4	−0.22	0.99	21	*
P3	0.43	0.20	39	0.51	P3	−0.48	0.70	20	*
Pz	0.75	0.66	47	0.97	Pz	−0.21	0.90	18	*
P4	0.45	0.51	45	0.85	P4	−0.32	0.69	17	*
O1	0.32	0.81	31	0.20	O1	−0.17	1.70	11	**
O2	0.82	0.76	43	0.73	O2	0.09	1.53	16	*

Group differences were tested using the Mann-Whitney U Test. Asterisks indicate significant group differences (* for p < 0.05. ** for p < 0.01).

**Table 3:** Matrix of correlation coefficients for behavioural RT and ERP data (Cz). Each index is calculated as AV–AO.

	Language group	N1 peak amplitude	P2 peak amplitude	N1 latency	P2 latency
Language group	1.000				
N1 peak amplitude	−0.047	1.000			
P2 peak amplitude	−0.631	0.251	1.000		
N1 latency	0.163	−0.008	−0.405	1.000	
P2 latency	−0.549	0.095	0.464	0.026	1.000
Behavioural RT	−0.700	0.066	0.700	−0.396	0.329
