# Supplementary material for: Language/Culture Modulates Brain and Gaze Processes in Audiovisual Speech Perception
Source: Sci Rep. 2016 Oct 13;6:35265. doi: 10.1038/srep35265 (PMC5062344; doi:10.1038/srep35265)
Supplement: Supplementary Information [file srep35265-s1.pdf]

Supplementary Information

Language/Culture Modulates Brain and Gaze Processes in Audiovisual Speech Perception

Satoko Hisanaga, Kaoru Sekiyama, Tomohiko Igasaki, and Nobuki Murayama

Checking for outliers – ERP peaks

To confirm that group effects were not driven by outliers, we checked for outliers in the mean latency and amplitude for both N1 and P2. Fig. S1 shows individual ERPs (event-related potentials) overlaid for each group. Individually determined N1/P2 values that were used for analyses are also shown in Table S1. We used the mean±2.5 SD as the criterion for non-outliers. None of the participants were outliers.

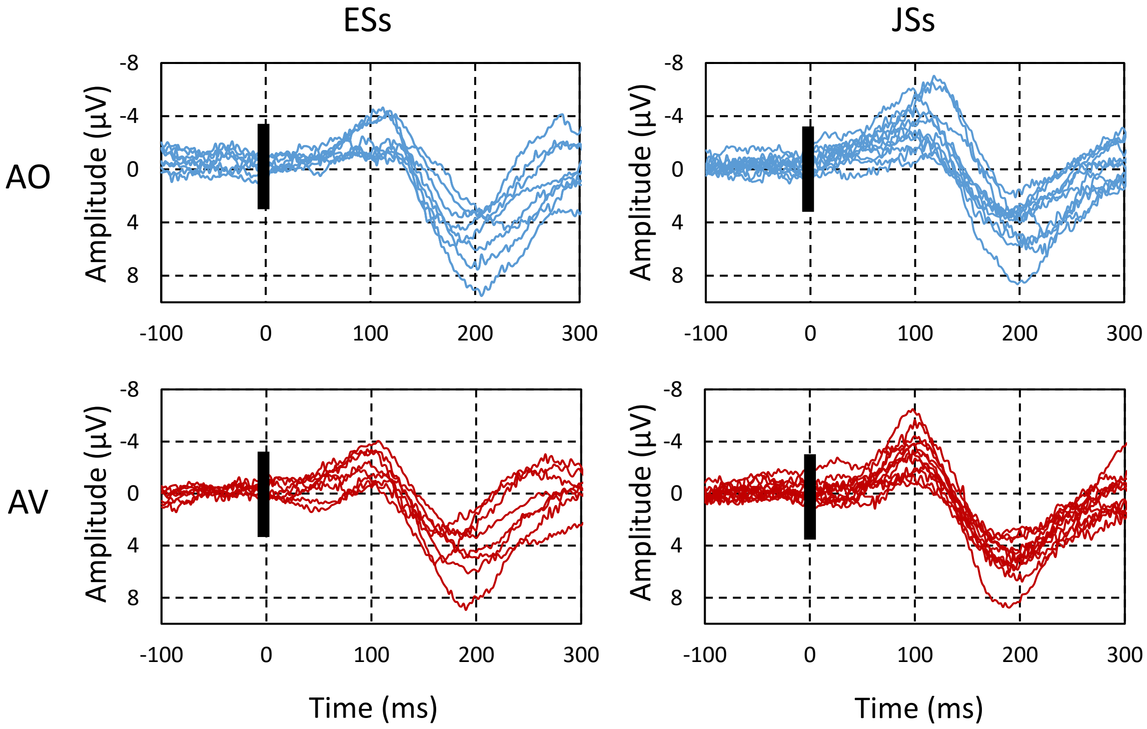

Fig. S1　ERP data of each individual for each condition and group.

Table S1. N1 and P2 latencies and amplitudes for each individual in each condition.

Group	Condition	ID	Latency (ms)		Amplitude (μV)	
			N1	P2	N1	P2
ESs	AO	S01	116.5	216.0	−4.14	3.80
		S02	98.0	182.0	−4.64	5.72
		S03	127.0	205.5	−1.31	3.59
		S04	99.0	188.0	−2.54	4.53
		S05	105.0	199.5	−1.66	5.99
		S06	121.0	220.0	−1.88	4.39
		S07	108.0	201.5	−3.96	9.51
		S08	122.5	211.0	−2.02	7.53
		Mean	112.13	202.94	−2.77	5.63
		SD	11.13	13.14	1.29	2.03
		Mean+2.5SD	84.31	170.09	0.44	10.72
		Mean−2.5SD	139.94	235.79	−5.98	0.54
	AV	S01	105.0	196.0	−4.48	3.41
		S02	101.5	165.5	−3.30	5.27
		S03	97.5	183.0	−0.70	3.17
		S04	95.5	170.0	−1.83	3.22
		S05	115.5	194.5	−0.98	4.50
		S06	98.0	198.5	−2.31	5.11
		S07	100.0	197.5	−3.19	8.48
		S08	112.0	189.5	−1.70	6.12
		Mean	103.14	186.81	−2.31	4.91
		SD	7.21	12.83	1.28	1.80
		Mean+2.5SD	85.10	154.73	0.88	9.41
		Mean−2.5SD	121.15	218.90	−5.51	0.40
JSs	AO	S01	107.5	230.0	−4.35	4.67
		S02	101.5	203.5	−6.09	8.48
		S03	99.0	205.0	−3.09	5.73
		S04	112.5	187.0	−2.51	3.42

	S05	104.5	193.5	−4.74	3.44
	S06	116.0	225.0	−6.52	6.03
	S07	105.5	199.5	−2.90	5.84
	S08	117.5	196.0	−1.30	3.63
	S09	117.5	205.5	−2.12	3.90
	S10	111.5	208.5	−3.73	3.99
	S11	94.5	191.0	−1.74	3.44
	S12	122.0	199.5	−7.03	1.87
	Mean	109.13	203.67	−3.84	4.53
	SD	8.43	12.84	1.92	1.74
	Mean+2.5SD	88.05	171.58	0.96	8.88
	Mean−2.5SD	130.21	235.76	−8.64	0.19
AV	S01	92.5	210.5	−3.80	5.38
	S02	82.5	193.5	−5.19	8.61
	S03	100.5	197.5	−1.49	5.21
	S04	96.0	194.0	−3.20	4.48
	S05	98.0	197.5	−3.41	5.76
	S06	100.5	208.0	−5.65	6.76
	S07	97.5	197.5	−3.88	6.04
	S08	82.0	204.5	−0.93	3.70
	S09	116.5	202.5	−2.05	3.95
	S10	91.0	189.5	−2.86	5.31
	S11	100.5	199.5	−1.92	3.74
	S12	96.5	216.5	−4.60	4.96
	Mean	96.17	200.92	−3.25	5.33
	SD	9.06	7.81	1.47	1.40
	Mean+2.5SD	73.52	181.4	0.44	8.82
	Mean−2.5SD	118.81	220.43	−6.93	1.83
